# BAE-ViT: An Efficient Multimodal Vision Transformer for Bone Age Estimation

**DOI:** 10.3390/tomography10120146

**Published:** 2024-12-13

**Authors:** Jinnian Zhang, Weijie Chen, Tanmayee Joshi, Xiaomin Zhang, Po-Ling Loh, Varun Jog, Richard J. Bruce, John W. Garrett, Alan B. McMillan

**Affiliations:** 1 Department of Electrical and Computer Engineering, University of Wisconsin-Madison, Madison, WI 53706, USA; 2 Department of Computer Science, University of Wisconsin-Madison, Madison, WI 53706, USA; 3 Department of Pure Mathematics and Mathematical Statistics, University of Cambridge, Cambridge CB2 1TN, UK; 4 Department of Radiology, University of Wisconsin-Madison, Madison, WI 53706, USA; 5 Department of Medical Physics, University of Wisconsin-Madison, Madison, WI 53706, USA; 6 Department of Biomedical Engineering, University of Wisconsin-Madison, Madison, WI 53706, USA

**Keywords:** bone age regression, machine learning, vision transformer, gender embedding, multimodal data

## Abstract

This research introduces BAE-ViT, a specialized vision transformer model developed for bone age estimation (BAE). This model is designed to efficiently merge image and sex data, a capability not present in traditional convolutional neural networks (CNNs). BAE-ViT employs a novel data fusion method to facilitate detailed interactions between visual and non-visual data by tokenizing non-visual information and concatenating all tokens (visual or non-visual) as the input to the model. The model underwent training on a large-scale dataset from the 2017 RSNA Pediatric Bone Age Machine Learning Challenge, where it exhibited commendable performance, particularly excelling in handling image distortions compared to existing models. The effectiveness of BAE-ViT was further affirmed through statistical analysis, demonstrating a strong correlation with the actual ground-truth labels. This study contributes to the field by showcasing the potential of vision transformers as a viable option for integrating multimodal data in medical imaging applications, specifically emphasizing their capacity to incorporate non-visual elements like sex information into the framework. This tokenization method not only demonstrates superior performance in this specific task but also offers a versatile framework for integrating multimodal data in medical imaging applications.

## 1. Introduction

Bone age assessment is one of the most common radiological studies, and is essential for experts to determine growth disorders from differences between bone and chronological age. Historically, skeletal development was assessed manually from metacarpals, phalanges, and carpals in X-rays using techniques such as Greulich–Pyle [1] and Tanner–Whitehouse [2]. However, these methods rely heavily on radiologists’ experience, leading to massive time and labor costs.

Recently, automatic bone age estimation (BAE) using deep learning has demonstrated superior efficiency and accuracy. The existing literature may be categorized into two types: methods that focus on specific anatomical Regions of Interest (ROI-based) and those that analyze entire images (image-based methods). ROI methods first extract image features of the palm or local bone parts using experts [3] or deep networks [4], which are then used for BAE by convolutional neural networks (CNNs) [5], residual attention networks [6], or regression models [7]. Although ROI methods are accurate, feature extraction requires much manual labor. The multi-stage pipeline also has low prediction efficiency, limiting clinical applicability. Image-based methods directly feed images into CNNs [8,9,10,11], which is efficient for both training and testing. The winning solution for the RSNA Pediatric Bone Age Challenge 2017 [12] used an InceptionV3-based model with a mean absolute error (MAE) of 4.2 months.

Sex information is important for BAE [13], and is often concatenated to image features either directly [14] or after being converted to an embedding vector by linear layers [15]. The combined features are then fed into a multilayer perceptron (MLP) for prediction. Notably, this method does not permit interactions between sex and image information.

Machine learning techniques have also been used to enhance traditional methods of bone age assessment by automating feature analysis. Subtle skeletal features, such as cortical decalcification and microfractures in CT scans, have been analyzed using texture analysis and deep learning methods to refine age and sex detection [16]. Texture-based local binary patterns and spatial attention mechanisms have been integrated into deep residual networks, addressing challenges specific to ethnicity in datasets [17]. Multiscale imaging approaches have been employed to study skeletal aging by combining traditional radiography with advanced microscopy techniques [18], while MRI-based models have been developed as non-invasive alternatives for age estimation through textural feature extraction [19].

Recently, vision transformers (ViTs) [20,21,22] have shown great potential in computer vision. Unlike CNNs, characterized by locality, the self-attention module [23] in ViTs allows each pixel in the input image to interact with all other pixels. ViTs can further handle multi-source inputs by converting them into sequences of tokens, making them a popular choice for multimodal data [24,25,26].

To the best of our knowledge, there is no existing work exploring multimodal ViTs in BAE. In this paper, we design a multimodal vision transformer, BAE-ViT, that integrates both image and sex information more efficiently than CNNs, and demonstrate its improved performance on normal and challenging samples.

## 2. Materials and Methods

### 2.1. Dataset

We consider two datasets of left-hand radiographs. The first dataset is publicly available from the 2017 RSNA Pediatric Bone Age Challenge. There are 12,611 training images (6833 male, 5778 female), 1425 validation images (773 male, 652 female), and 200 test images (100 male, 100 female). Based on the study in [27], this dataset is distinguished as one of the largest and most comprehensive, extensively published and validated, which enhances the reproducibility and applicability of our research. For training and validation, images were sourced from the Children’s Hospital Colorado (Aurora, Colo) and Lucile Packard Children’s Hospital at Stanford, while the test set images were exclusively obtained from the Lucile Packard Children’s Hospital [12], making this a multi-site dataset. The second dataset is an external validation dataset consisting of data from our own institution from 100 different patients (61 male, 39 female). This retrospective study was approved by our Institutional Review Board. The need for informed consent was waived for this Health Insurance Portability and Accountability Act-compliant study.

### 2.2. Models

**Figure 1:**
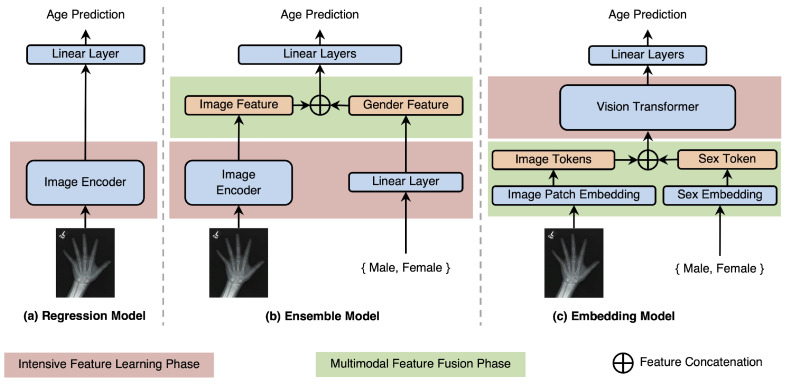
A comparison of architectures between the regression, ensemble, and embedding models. The red area indicates the intensive feature learning phase (red), which requires significant computational resources, while the green area indicates the multimodal feature fusion phase (green), where image features are integrated with non-visual features such as sex. (**a**) Regression model: This model only takes images as inputs, without using biological sex information. (**b**) Ensemble model: In this model, two branches encode the image and sex information into feature vectors separately. These features are concatenated and fed into linear layers for bone age estimation. The biological sex information is integrated after the image encoder and processed only by the linear layer. (**c**) Embedding model (proposed): Our proposed multimodal vision transformer converts both the image and sex information into tokens. These tokens interact in the transformer blocks through attention mechanisms and are finally projected through a linear layer for predictions.

Two traditional model architectures for BAE are shown in Figure 1a,b. The regression model only uses CNNs or ViTs to obtain image features and a linear layer for regression. Sex information is ignored. The ensemble model has two branches to encode image and sex information, which are concatenated as inputs to three linear layers for prediction. However, extracting features from different data types using independent branches may be suboptimal. Therefore, we convert image and sex information into tokens of the same dimension, which interact in the transformer blocks and are aggregated and fed into the linear regressor in Figure 1c. The hyperparameters used were those recommended by the original model designers, leveraging their extensive expertise and experimentation, while ensuring consistency and fairness in comparisons by selecting configurations with a similar number of parameters.

In our methodology, we integrate visual and non-visual data, leveraging the strengths of ViTs for their comprehensive data processing capabilities, a task for which the Swin Transformer and DeiT model’s distillation strategy are particularly suited. The Swin Transformer, customized for the nuanced processing of image data, and the DeiT’s efficiency-focused distillation approach, pave the way for TinyViT, a model that embodies the essence of rapid learning through progressive distillation from large, pretrained models. This model harmonizes performance with computational efficiency, a testament to its sophisticated design. Additionally, we employ an architectural innovation by utilizing inverted residual blocks, which adopt a narrow -> wide -> narrow channel structure, contrasting with traditional residual blocks. This choice is complemented by the incorporation of lightweight and efficient MBConv layers in the initial stages of our model, optimizing the learning of low-level representations through their strong inductive biases, thereby enhancing both the efficiency and effectiveness of our integrated approach.

From a methodological perspective, tokenization is a process that transforms multimodal data into uniform vectors, facilitating cross-validation among features from diverse modalities. Typically, features from images are extracted through convolutional blocks, while sex information is derived using linear layer projection. The specific technical details are elaborated upon in the following sections.

**Figure 2:**
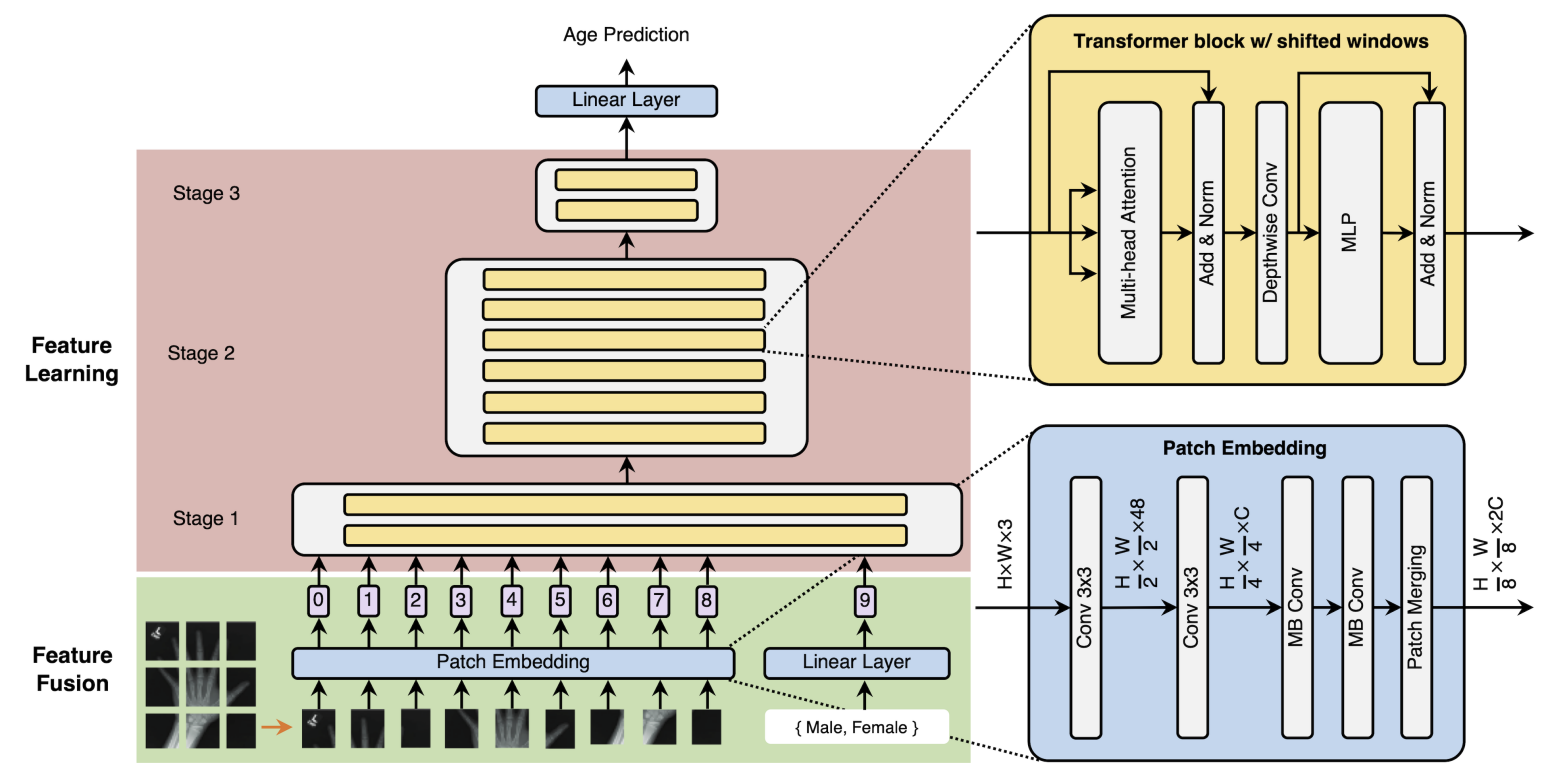
BAE-ViT architecture with biological sex embedding. This diagram illustrates the three-stage design of the BAE-ViT architecture. In the feature fusion phase (green), the model uses patch-embedding, convolutions, and MBConv blocks, followed by patch merging to create token sequences. The biological sex information is tokenized through a linear layer and processed by the transformer alongside other visual patch tokens. In the feature learning phase (red), stages 1, 2, and 3 involve transformer blocks with shifted window attention, processing features at varying scales. The architecture is designed for efficient feature extraction and integrates sex information, facilitating enhanced classification performance.

Our proposed multimodal ViT architecture is illustrated in Figure 2. We follow the design of TinyViT-21M [28], which is more efficient than DeiT-S [21] and Swin-T [20], and has even better performance than Swin-L on ImageNet-1k [29]. It consists of a patch-embedding layer, patch merging layers, and transformer blocks with shifted windows. Additionally, we generate a novel sex embedding using a linear layer with 2C hidden nodes (the dimension of the image tokens).

Considering the default input dimension for a visual transformer, the image is initially resized or randomly cropped to dimensions of 224×224 pixels. We obtain the image tokens through the patch-embedding layer, involving two 3×3 convolutional layers with stride 3 to reduce the input resolution from (H,W) to (H/4,W/4) and increase the channel size from 3 to C. After two inverted residual blocks (also known as MBConv) [30], we use the patch-merging layer to decrease the resolution of feature maps to (H/8,W/8), in which there are two pointwise convolutional layers and one depthwise convolutional layer in the middle with kernel size 3 and stride 2. Finally, the feature maps are rearranged to HW/64 image tokens with dimension 2C. Combined with the sex token, all HW/64+1 tokens are fed into the transformer blocks.

Traditional transformer blocks contain multi-head attention [23], layer normalization [31], and MLP. However, the global computational complexity is quadratic in the number of tokens N. To improve efficiency, TinyViT introduces shifted windows from Swin-T [20] to separate image tokens into groups. Attention is performed within each group:

(1) $$\mathrm{Attention}\left(Q,K,V\right)=\mathrm{softmax}\left(QK^{T}/\sqrt{d}+B\right)V,$$

where $Q,K,V\in R^{M^{2}\times d}$ are the query, key, and value matrices; *d* is the hidden dimension; and $M^{2}$ is the group size. *B* is the relative position bias matrix taken from a learnable matrix $\hat{B}\in R^{\left(2M+1)\times (2M+1\right)}$ according to the relative position of each token pair in each axis, lying in [−M+1,M−1]. This reduces the computational complexity to $O(NM^{2})$. Notably, the group partition is shifted by half the group size in adjacent blocks to avoid performance degradation due to lack of interaction across groups. Moreover, a depthwise convolution with kernel size 3 and stride 1 is applied to capture local features to supplement global attention. In BAE-ViT, the sex token is included in each group for interaction. Therefore, *Q*, *K*, *V* have dimensions $(M^{2}+1)\times d$ in the attention module of transformer blocks. We fix the last row and column in $B\in R^{(M^{2}+1)\times d}$ to 0, indicating no relative positional bias in the sex token. We did not observe performance improvements after introducing relative positional bias.

### 2.3. Evaluation

We compare BAE-ViT to regression and ensemble models using three different CNNs (InceptionV3 [32], ResNet50 [33], and EfficientNet-B5 [34]) and TinyViT as the image encoder, after removing their original classification head. In the ensemble model, we fix the size of the sex feature vector as 8 and the hidden dimension of the first two linear layers as 1000. The last linear layer is a single neuron.

For model evaluation, we calculated the MAE between model predictions and the ground truth. Each test image was resized to make the shorter edge equal to the input resolution, and we obtained a square crop of the resized image at the center along the longer edge. We also adopted a multi-crop test method from [35]: for each test image, we obtained 10 random square crops with the given resolution and fed them into the model. The median of the outputs was used as the final prediction. The number of random crops needs to be sufficiently large to yield stable predictions. The adoption of a multi-crop testing methodology, specifically the utilization of 10 random square crops, represents a standard and widely recognized approach in the evaluation of deep learning models, particularly in the domain of image classification. This methodological choice is predicated on the objective of enhancing model robustness and generalization by simulating a diverse array of real-world imaging conditions. By incorporating crops from the four corners and center of the image, along with their horizontal flips, this approach systematically evaluates the model’s performance across varied spatial presentations, thereby mimicking the multifaceted nature of real-world visual perception. Such a strategy not only aims to mitigate potential overfitting by challenging the model with less predictable image segments but also leverages ensemble learning principles, wherein the aggregation of predictions across these crops serves to bolster prediction accuracy and stability. This comprehensive evaluation framework thus ensures that the model’s high performance is indicative of genuine learning rather than mere memorization of training data biases. Despite the increased computational demands associated with processing multiple crops per image, the adoption of this popular and standardized method is justified by its significant contributions to improving the depth and reliability of model evaluations, thereby facilitating the development of more adaptable and dependable image classification models.

To evaluate the influence of demographic attributes on the model evaluation, we designed a label perturbation experiment during the testing stage. In this experiment, biological sex labels in the RSNA dataset were intentionally perturbed by assigning incorrect values. This altered demographic information was then used to assess model performance on X-ray scans, allowing us to observe the impact of such inconsistencies on prediction accuracy.

In our study, we employ ScoreCAM [36] for generating class activation maps due to its distinct advantages over gradient-based methods like Grad-CAM [37]. ScoreCAM’s reliance on forward-pass activations, rather than gradients, offers clearer and more interpretable visual explanations of model decisions by directly assessing the impact of different image regions on the model’s output. This approach mitigates issues associated with gradient-based visualizations, such as noisy gradients, providing a more accurate reflection of the model’s focus areas. Although ScoreCAM demands higher computational resources due to its need for multiple forward passes, its capacity for producing more faithful and interpretable activation maps justifies its selection in our methodology, enhancing the transparency and accuracy of our model evaluations.

In our analysis, the Wilcoxon signed-rank test was employed to identify statistically significant differences between model predictions and ground-truth labels, with a predefined significance level set at 0.05. This choice reflects standard practice in statistical analysis, balancing the detection of genuine effects against the risk of false discoveries. To address the concern of increased type I errors due to multiple comparisons, the Bonferroni correction method was rigorously applied, thereby adjusting the significance thresholds to a more stringent criterion. This step is crucial in preserving the integrity of our findings amid multiple statistical tests. It is pertinent to note, however, that while the Wilcoxon signed-rank test is robust to the non-normality of data, it operates under the assumption that the differences between matched pairs are symmetrically distributed around the median. This assumption is a potential limitation of the method, suggesting cautious interpretation of results when the distribution of differences is expected to deviate from symmetry. Our adherence to these statistical protocols underscores our commitment to methodological rigor and the reliability of our conclusions, within the acknowledged constraints of the chosen statistical tests.

## 3. Results

All models were trained on the RSNA training dataset; the RSNA validation dataset was used to optimize the hyperparameters. The trained models were evaluated on both the RSNA and external datasets. All experiments were conducted on two NVIDIA A100 GPUs (NVIDIA Corporation, Santa Clara, CA, USA) with 80 GB memory using PyTorch (software version 1.7.1, created by Meta Platforms, Inc., Menlo Park, CA, USA). For image preprocessing, we obtained the mean and standard deviation for z-score normalization based on the RSNA dataset. Data augmentation techniques included RandAugment [38] and random erasing [39]. For training, we inherited the hyperparameters from Swin-T: when using CNNs as image encoders, we set the learning rate to $2.5\times 10^{-4}$, batch size to 256, and number of epochs to 500. All models were trained from scratch. For the ensemble model that uses TinyViT, we used the same settings. The dropout rate for all ensemble models was 0.5. For the regression model with TinyViT as the image encoder, we used a learning rate of $3.1\times 10^{-5}$, batch size of 32, and number of epochs of 300. Weights pretrained on ImageNet-1k were used for initialization. The training settings for BAE-ViT were the same as the regression model with TinyViT.

### 3.1. Comparison of Performance of CNNs and ViTs

Table 1 summarizes the performance of our proposed multimodal BAE-ViT and the regression and ensemble models with CNNs and ViTs as the image encoder. We observe that sex information is critical for BAE: the ensemble model outperforms the regression model by more than 1.0 month in MAE. This conforms to the expectation that models are prone to fitting the average age of images with similar skeletal maturity, but since females generally achieve maturity nearly two years earlier than males, models that disregard sex information have larger errors.

We found that using separate sex-specific models led to better performance than using a single model. The regression model for males always had a lower MAE than that of females on the RSNA test dataset, but the opposite was true on the external test dataset. This suggests that distribution shifts exist between the training and test datasets. Compared to the ensemble model, the average performance of separate models was slightly better when using Inception-V3, EfficientNet-B5, or TinyViT as the image encoder. A possible reason is that simply concatenating image and sex features is inefficient, so performance depends on the architecture of the image encoder. For example, when using ResNet50, the ensemble model achieved the best MAE on the RSNA dataset.

Our proposed BAE-ViT achieved the best performance on both the RSNA and external datasets with the multi-crop test method. When using only the center crop of test images, BAE-ViT was only 0.1 worse in MAE than the ensemble model with ResNet50 on the RSNA dataset. However, it performs much better on the external dataset, showing better generalization. This demonstrates that the interaction between image and sex information by using attention in transformer blocks is more efficient than the ensemble model.

### 3.2. Visualization of Heatmaps

**Figure 3:**
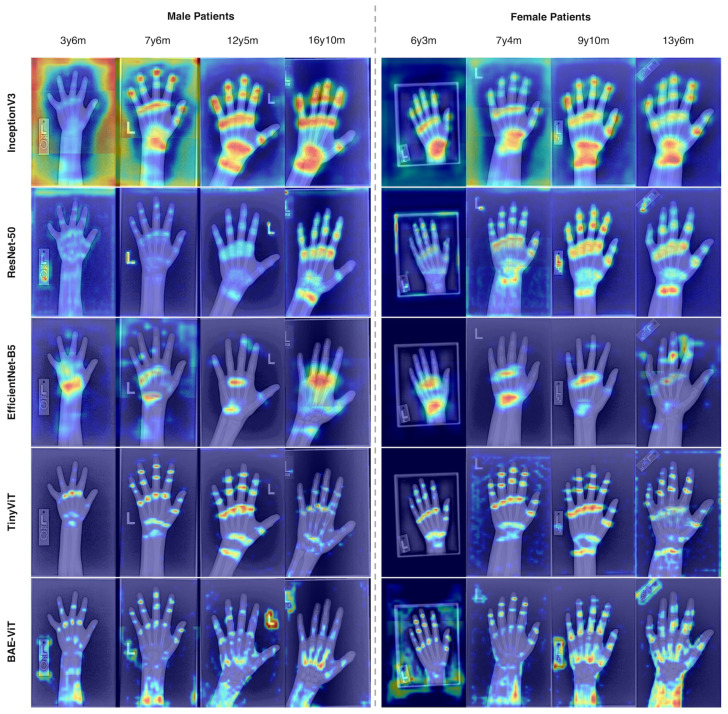
Heatmaps of our proposed BAE-ViT and ensemble models using CNNs or TinyViT as the image encoder by ScoreCAM. The left four columns are male with bone ages of 34.2, 89.9, 149.1, and 202.3 months, respectively. The right four columns are female with bone ages of 75.0, 87.5, 118.2, and 162.0 months, respectively. Heatmaps tend to highlight joints within the fingers and hand.

We generate heatmaps for different age groups using ScoreCAM in Figure 3. Compared to CNNs, that have coarser receptive fields, ViTs can focus on key local regions due to the global attention of transformer blocks. Note that some models fail to attend to the correct region for images with a bone age less than 100 months, e.g., the ensemble model with Inception-V3 as the image encoder also attends to the border of images with a bone age of 34.2 (first column), 87.5 (sixth column), and 89.9 (second column). This may be due to a lack of training samples in this age group. Moreover, since sex information was used in all models, there is no obvious difference between the heatmaps for males and females. Notably, our proposed BAE-ViT additionally attends to the radius and ulna, which will be the focus of future research.

### 3.3. Performance of Models in Different Age Groups

**Figure 4:**
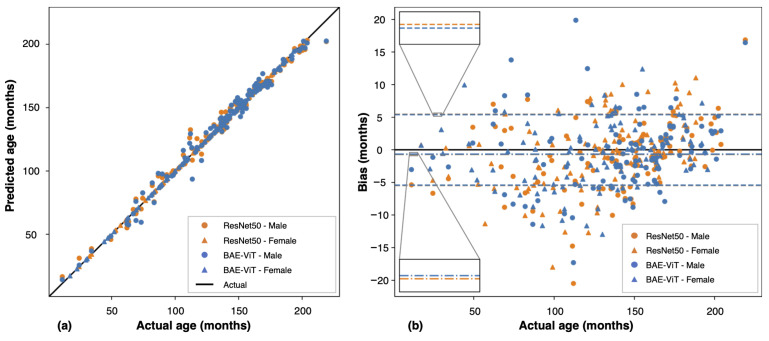
Performance comparison between our proposed BAE-ViT model and the ResNet50 ensemble model. (**a**) Correlation between actual and predicted bone ages, demonstrating high correlation for both models. (**b**) Mean bias and standard deviation of bias for the models, with dot-dashed lines representing mean bias and dashed lines indicating the standard deviation. The mean bias, defined as the signed average error, indicates whether the model’s predictions are consistently higher or lower than the true values. Both models exhibit mean bias values close to zero (−0.66 for BAE-ViT and −0.70 for ResNet50), suggesting no significant overestimation or underestimation. BAE-ViT shows a slightly lower mean bias and standard deviation (5.40) compared to the ResNet50 ensemble model (5.50 standard deviation), indicating its superior accuracy and consistency in predictions.

Our BAE-ViT achieves the highest performance on the RSNA test dataset, with an average MAE of 4.1 s using the multi-crop test. The male MAE is 4.4 months and the female MAE is 3.9. Its predictions show a high agreement with the labels Figure 4a, with a mean bias of −0.66 and a standard deviation of 5.40 months (Figure 4b), slightly better than the ensemble model with ResNet50 (mean bias of −0.7 and standard deviation of 5.50 months). Based on a Wilcoxon signed-rank test, the statistical results showed no significant differences between model predictions and labels (*p*-values of ResNet50 and BAE-ViT are 0.2 and 0.4, respectively). Overall, BAE-ViT is accurate and reliable.

### 3.4. Training Techniques for Ensemble Models

**Figure 5:**
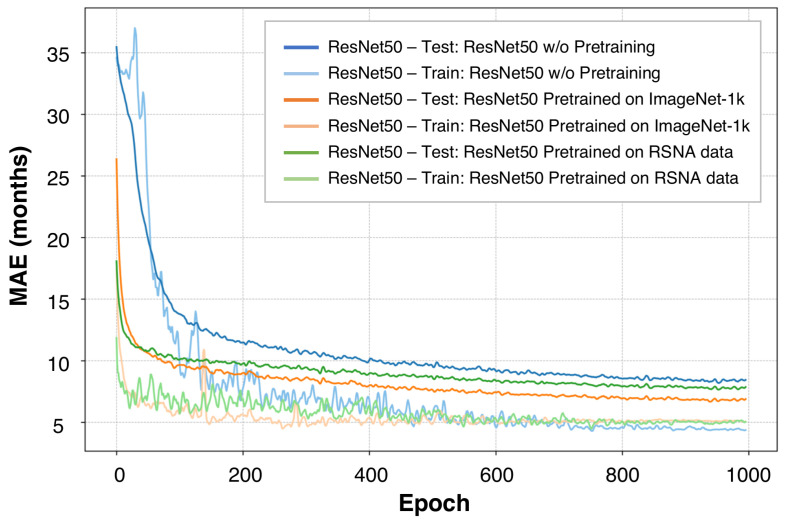
ResNet50 training and testing loss comparison. This figure presents the loss curves for a ResNet50 model under different training conditions. Lighter solid lines show the training loss, and darker dashed lines represent the testing loss. The graph compares end-to-end training with pretraining on ImageNet-1k and RSNA datasets. The lower testing loss across all methods could imply a limited diversity in the testing dataset compared to the training dataset.

We can train the ensemble model either from scratch or using a two-phase method, where we first pretrain the image encoder, and then train the model with the weights of the image encoder fixed or variable. This should intuitively lead to better performance. However, the results in Table 2 show that training from scratch yields a lower MAE than the two-phase method for different models as the image encoder. We investigate the curves of both training and test error, as shown in Figure 5. Although pretrained weights can accelerate the convergence rate, they lead to overfitting. Hence, the model is more sensitive to training hyperparameters.

For two-phase methods with a fixed image encoder, it is noteworthy that pretraining the image encoder on ImageNet-1k results in an MAE exceeding 4.0 months compared to pretraining on RSNA data. This discrepancy is likely due to the domain gap between ImageNet-1k and bone images. In contrast, for two-phase methods with a variable image encoder, the final MAE is closer between pretraining on the ImageNet-1k and RSNA datasets, indicating a reduced impact of the pretraining domain on the model’s performance given the variable image encoder weights. Moreover, fixing the pretrained weights of the image encoder is generally less efficient. Therefore, even after pretraining, the image encoder needs to adjust the weights for better interaction with the sex branch to improve accuracy.

### 3.5. Robustness of Models over Bad Examples

We evaluate the robustness of BAE-ViT and different ensemble models over 44 selected images from different patients deemed to be of poor quality, e.g., containing multiple hands, the right hand, or bad positioning. Radiographers often need to retake poor-quality images, expending more resources. However, our proposed BAE-ViT shows better robustness on these bad examples than ensemble models, with only a 9.9 month MAE. Therefore, one can improve efficiency by avoiding image reacquisition even in the presence of low-quality data.

### 3.6. Sensitivity to Demographic Label Perturbation

The label perturbation experiment revealed a substantial performance degradation when incorrect biological sex labels were provided. Specifically, the mean absolute error (MAE) increased dramatically from 4.1 months to 20.9 months with center cropping and from 4.2 months to 21.5 months with multi-cropping. These results underscore the sensitivity of the model to accurate demographic information.

## 4. Discussion

We have designed a new architecture to combine different types of data more efficiently in bone age regression. Due to their inherent architectural limitations, CNNs struggle to process multimodal data directly. Traditional methods include converting the sex information to an additional channel of the input image, which is highly inefficient, or using an ensemble model to concatenate image features from CNNs and sex features from another branch, which does not allow non-visual features to interact with vision features. However, by leveraging properties of vision transformers, our multimodal BAE-ViT facilitates the encoding of non-visual attributes within its transformer blocks, thereby enabling intricate interactions between image-based and non-visual information. This capability paves the way for the integration of additional data modalities, such as demographic distributions, into a unified network architecture. Such an approach holds promise for enhancing both the performance and interpretability of the model.

### 4.1. Model Comparison

Several recent studies have presented advancements in bone age estimation techniques. For instance, a study [40] utilized CNNs combined with the Tanner–Whitehouse method to extract phalangeal and carpal bone features for predicting bone age. This study employed an Extreme Learning Machine (ELM) algorithm [41], achieving an MAE of 6.1 months on the RSNA dataset, but did not incorporate sex information. Another study [42] implemented a CNN model to estimate bone age from metacarpal and carpal bones, reporting MAEs of 5.6 and 6.0 months on a private dataset of 3871 children. A further study [43] leveraged the YOLOv5 network to extract region-of-interest (ROI) patches, incorporating biological sex information by passing it through linear and softmax layers to align with the patch dimensions. This information was then used to modulate ROI patches through a Swin Transformer network, achieving an MAE of 4.6 months on the RSNA dataset.

In contrast, our proposed method utilizes entire X-ray scans and introduces a novel approach by converting non-visual information (e.g., biological sex) into a token. This token undergoes attention computation with visual tokens, allowing for a generalized application to other data formats (e.g., demographic, audio, image, text) through tokenization. Our approach achieved an MAE of 4.1 months on the RSNA dataset. Furthermore, we generate heatmaps by using ScoreCAM to better understand the behavior of CNNs and ViTs in Figure 3. We also investigate different training techniques for ensemble models, and observe that pretraining the image encoder leads to overfitting, and hence worse performance. Additionally, we evaluate BAE-ViT and ensemble models on challenging examples and find that our proposed model is more robust to distortions in images in Table 3.

### 4.2. Limitations

This work is not without limitations. First, we only evaluated models for one task, using a publicly available dataset. It would be interesting to see if our proposed BAE-ViT can also outperform the other models on similar datasets that contain both image and sex information, such as the UTKFace dataset [44], that is popular in age regression tasks. However, the RSNA dataset is one of the largest bone age regression datasets and has been widely studied in the literature. Our work is therefore still useful and can serve as a strong baseline for comparison in the future. Second, we only evaluate the robustness of models on a few challenging examples. There are more types of distortions in bone images in practice, and it is important to investigate if our proposed model is still resistant to them. Third, the ensemble models we studied primarily focus on combining different types of input data. However, ensemble learning also encompasses integrating multiple models with varying architectures that process the same inputs. Models with heterogeneous architectures may possess distinct strengths in handling different aspects of the input data, potentially leading to improved overall performance. This approach has been shown to be effective, as demonstrated in the RSNA Challenge [45], and is widely adopted in medical imaging. Therefore, it is crucial to investigate whether our proposed BAE-ViT can maintain its superior performance when integrated into an ensemble framework with heterogeneous models. It is notable that the ensemble method can also be applied to our proposed model.

### 4.3. Clinical Implementation

Potential challenges when implementing this model in a real clinical setting include the requirement for high computational power and integration with existing hospital systems. Although TinyViT is a knowledge distillation model with high performance and fewer parameters, it still requires a GPU for inference. There currently exist hundreds of FDA-approved AI software tools in radiology. Some tools utilize local inference, others utilize cloud providers. The allocation and availability of GPU resources does not appear to be preventing the utilization of AI in radiology. BAE-ViT does not have excessively large computational needs. Integration into hospital systems: Additional work would be needed to fully integrate any AI solution into radiology practice. This was not a major focus of our work. There exist several solutions that provide AI orchestration across various vendor platforms, for example, NVIDIA Clara, but many other competitors already exist in the field.

In the present study, our primary target has been on the regression analysis of bone age, utilizing a limited set of two distinct types of input data. Expanding the diversity of input data sources is anticipated to more comprehensively elucidate the advantages of ViT architectures over CNNs in the field of medical imaging. In conclusion, leveraging self-supervised pretraining techniques on large-scale public datasets has been shown to potentially improve the performance of ViT models [22]. From a clinical perspective, enhancing the interpretability of these machine learning models by providing detailed insights in the intermediate computational stages and robustness analysis could bolster radiologists’ confidence in the automated system.

### 4.4. Impact of Demographic Label Integrity

The label perturbation experiment underscores the critical role of accurate demographic data in our model’s performance. The significant performance drop observed with incorrect labels is likely attributable to the early fusion approach employed in our model. In this design, the biological sex attribute is first converted into a unified token, which later contributes to the feature learning phase (Shown in Figure 1c). This integration allows the model to observe specific correlations between visual features and sex attributes, enabling it to learn both shared and distinct features. However, incorrect labels disrupt this process, impairing feature extraction and leading to erroneous predictions.

In real-world healthcare scenarios, such mismatches in demographic labels are rare, as robust data validation protocols and regulatory standards typically minimize such errors. Nevertheless, our findings underscore the importance of maintaining data integrity in automated systems. Addressing these vulnerabilities further could involve incorporating mechanisms to detect and mitigate the impact of label inconsistencies, enhancing both robustness and reliability in clinical applications.

## 5. Conclusions

In this study, we designed a vision transformer model for bone age estimation, named BAE-ViT, which enables interactions between image and sex information via a tokenization method within transformer blocks. BAE-ViT demonstrated superior performance by achieving a lower mean absolute error (MAE) compared to traditional models and exhibited greater robustness when handling degraded or poor-quality images. This highlights the model’s potential in improving bone age estimation accuracy and its applicability in challenging imaging conditions.

## Figures and Tables

**Table 1:** Performance comparison between our proposed BAE-ViT and the regression and ensemble models that use CNNs and ViTs as the image encoder. For regression models, we consider not only a single model for all images (Regression), but also two models for male images and female images separately (Regression-S). For Regression-S, we show the average MAE on both the whole test dataset and the male and female test data in months. The best performance is shown in bold.

Model		Param. No. (M)	Input Res. (px)	Sex (Y/N)	RSNA MAE (↓)		External MAE (↓)	
					Center	Multi-Crop	Center	Multi-Crop
RSNA Challenge winner		>24	500^2^	Y	-	4.2	-	-
Ensemble-VGG 16		>138	600^2^	Y	-	-	8.8	-
Inception-V3	Regression	25	500^2^	N	6.1	5.7	8.1	8.0
					4.6	4.2	7.2	7.5
	Regression-S	25	500^2^	-	M: 4.4	M: 4.0	M: 7.4	M: 7.5
					F: 4.7	F: 5.0	F: 6.9	F: 7.4
	Ensemble	25	500^2^	Y	4.8	4.4	7.1	7.0
ResNet50	Regression	24	500^2^	N	6.8	6.7	8.6	8.4
					5.0	4.5	7.6	7.7
	Regression-S	24	500^2^	-	M: 4.6	M: 4.3	M: 7.7	M: 7.7
					F: 5.3	F: 4.7	F: 7.4	F: 7.6
	Ensemble	24	500^2^	Y	**4.3**	4.2	7.1	7.2
EfficientNet-B5	Regression	28	456^2^	N	6.6	5.7	8.1	8.0
					5.4	4.9	7.2	7.1
	Regression-S	30	456^2^	-	M: 4.9	M: 4.5	M: 7.5	M: 7.1
					F: 5.8	F: 5.3	F: 6.7	F: 6.9
	Ensemble	30	456^2^	Y	5.5	4.9	7.4	7.2
TinyViT	Regression	21	500^2^	N	6.0	5.6	8.4	7.8
					4.6	4.4	7.0	7.1
	Regression-S	21	512^2^	-	M: 4.5	M: 4.5	M: 7.2	M: 7.2
					F: 4.7	F: 4.3	F: 6.7	F: 6.8
	Ensemble	21	512^2^	Y	4.9	4.7	6.9	7.0
BAE-ViT		21	512^2^	Y	4.4	**4.1**	**6.7**	**6.9**

**Table 2:** MAE scores in months for ensemble models with different image encoders, comparing end-to-end and two-phase training with fixed and non-fixed weights.

Models	End-to-End	Pretrained on ImageNet-1k		Pretrained on RSNA Data	
		Fixed	Non-Fixed	Fixed	Non-Fixed
Inception-V3	4.8	9.5	5.2	5.3	4.9
ResNet50	4.5	9.7	5.1	5.8	5.2
TinyViT	4.9	9.4	5.8	5.2	5.8

**Table 3:** Evaluation of BAE-ViT against various ensemble models using different image encoders on challenging examples with image distortions, measured by MAE. The table compares performance under center-crop and multi-crop test conditions. The lowest MAE, indicating the best performance, is in bold, underscoring the robustness of the proposed BAE-ViT model to image distortions.

Test Method	Center-Crop	Multi-Crop
Inception-V3	10.7	10.5
ResNet50	10.6	10.8
EfficientNet-B5	11.3	11.2
TinyViT	10.3	10.5
BAE-ViT	**9.9**	**10.1**

## Data Availability

The primary dataset supporting this study, consisting of 14,236 cases, is accessible through the 2017 RSNA Pediatric Bone Age Challenge, available at https://www.rsna.org/education/ai-resources-and-training/ai-image-challenge/rsna-pediatric-bone-age-challenge-2017 (accessed on 5 November 2023). A supplementary dataset comprising 100 cases, obtained in compliance with institutional protocols, is not available for public access due to privacy and ethical restrictions. The code is available at https://github.com/mimrtl/BAE_ViT (accessed on 5 November 2023).
